# Alveolar Epithelial Denudation Is a Major Factor in the Pathogenesis of Pleuroparenchymal Fibroelastosis

**DOI:** 10.3390/jcm10050895

**Published:** 2021-02-24

**Authors:** Yoshiaki Zaizen, Yuri Tachibana, Yukio Kashima, Andrey Bychkov, Kazuhiro Tabata, Kyoko Otani, Yoshiaki Kinoshita, Yasuhiko Yamano, Kensuke Kataoka, Kazuya Ichikado, Masaki Okamoto, Tomoo Kishaba, Remi Mito, Koichi Nishimura, Mari Yamasue, Kazuki Nabeshima, Kentaro Watanabe, Yasuhiro Kondoh, Junya Fukuoka

**Affiliations:** 1 Department of Pathology, Nagasaki University Graduate School of Biomedical Sciences, 1-7-1 Sakamoto, Nagasaki 852-8501, Japan; zaizen_yoshiaki@med.kurume-u.ac.jp (Y.Z.); y.tachibana19890221@gmail.com (Y.T.); bychkov.andrey@kameda.jp (A.B.); 2 Division of Respirology, Neurology and Rheumatology, Department of Medicine, Kurume University School of Medicine, 67 Asahi-machi, Kurume, Fukuoka 830–830-0011, Japan; okamoto_masaki@med.kurume-u.ac.jp; 3 Department of Pathology, Hyogo Prefectural Awaji Medical Center, 1-1-137 Shioya, Sumoto, Hyogo 656-0021, Japan; yksmn0504@yahoo.co.jp; 4 Department of Pathology, Kameda Medical Center, 929 Higashi-cho, Kamogawa, Chiba 296-8602, Japan; 5 Division of Pathology, Department of Oncology, Kagoshima University Graduate School of Medical and Dental Sciences, 8-35-1 Sakuragaoka, Kagoshima 890-8544, Japan; tabatak.kufm@gmail.com; 6 Department of Pathology, Yodogawa Christian Hospital, 1-7-50 Kunijima, Higashiyodogawa-ku, Osaka 533-0024, Japan; o.kyoko303@gmail.com; 7 Department of Respiratory Medicine, Fukuoka University Chikushi Hospital, 1-1-1 Zokumyouin, Chikushino, Fukuoka 818-8502, Japan; y3kinoshita@gmail.com; 8 Department of Respiratory Medicine and Allergy, Tosei General Hospital, 160 Nishioiwake, Seto, Aichi 489-8642, Japan; yaya0630g@gmail.com (Y.Y.); kataoka@tosei.or.jp (K.K.); konyasu2003@yahoo.co.jp (Y.K.); 9 Department of Respiratory Medicine, Saiseikai Kumamoto Hospital, 5-3-1 Chikami, Minami-ku, Kumamoto 861-4193, Japan; kazuya-ichikado@saiseikaikumamoto.jp; 10 Department of Respiratory Medicine, Okinawa Chubu Hospital, 281 Miyazato, Uruma, Okinawa 904-2293, Japan; kishabatomoo@gmail.com; 11 Department of Respiratory Medicine, Kumamoto University Hospital, 1-1-1 Honjou, Chuou-ku, Kumamoto 860-8556, Japan; candypinkcolor@yahoo.co.jp; 12 Department of Respiratory Medicine, National Center for Geriatrics and Gerontology, 7-430 Morioka-chou, Oobu, Aichi 474-8511, Japan; koichi-nishimura@nifty.com; 13 Department of Respiratory Medicine, Oita University Hospital, 1-1 Idaigaoka, Hasamamachi Yufu, Oita 879-5593, Japan; sai-mari@oita-u.ac.jp; 14 Department of Pathology, Fukuoka University School of Medicine and Hospital, 7-45-1 Nanakuma, Jonan-ku, Fukuoka 814-0180, Japan; kaznabes@fukuoka-u.ac.jp; 15 Department of Respiratory Medicine, Nishi Fukuoka Hospital, 3-18-8 Ikinomatsubara, Nishi-ku, Fukuoka 819-8555, Japan; watanabe@fukuoka-u.ac.jp

**Keywords:** pathology, pleuroparenchymal fibroelastosis, idiopathic pulmonary fibrosis, epithelial denudation, epithelial detachment, image analysis, classification, pathogenic mechanism

## Abstract

The pathogenesis of pleuroparenchymal fibroelastosis (PPFE), a rare interstitial lung disease, remains unclear. Based on previous reports and our experience, we hypothesized that alveolar epithelial denudation (AED) was involved in the pathogenesis of PPFE. This multicenter retrospective study investigated the percentage of AED and the features of the denudated areas in 26 PPFE cases, 30 idiopathic pulmonary fibrosis (IPF) cases, and 29 controls. PPFE patients had lower forced vital capacities and higher residual volume/total lung capacities in pulmonary function tests compared to IPF and control patients. Histopathologically, subpleural fibroelastosis was observed in PPFE, and AED was observed in 12.01% of cases in the subpleural or interlobular septa regardless of fibroelastosis. The percentage of AED in the PPFE group was significantly higher than that in the IPF group (6.84%; *p* = 0.03) and the normal group (1.19%; *p* < 0.001). In the IPF group, the percentage of AED and the presence of PPFE-like lesions in the upper lobes were examined radiologically, but no correlation was found. We showed that AED frequently occurred in PPFE. AED was less frequent in IPF, which, in combination with imaging data, suggests that PPFE may have a different pathogenesis from IPF.

## 1. Introduction

Pleuroparenchymal fibroelastosis (PPFE) is a rare type of interstitial lung disease characterized by the formation of lesions in the upper lobe of the lung [1,2,3]. Histopathologically, PPFE is characterized by fibroelastosis, predominantly in the upper lobe, and a fibrotic lesion that is characterized by an aggregation of elastic fibers in the subpleural zone [2,3,4,5]. These histopathological characteristics of PPFE are different from the usual interstitial pneumonia (UIP) pattern, which is the pathological pattern of idiopathic pulmonary fibrosis (IPF) characterized by architectural distortion with dense collagenous fibrosis, including honeycombing in the lower lobe of the lung [4,5]. While PPFE is usually considered an idiopathic disease, some specific causes were also reported, such as bone marrow and postlung transplant graft-versus-host disease (GVHD) [6,7], fungal infections such as those caused by *Aspergillus* [5,8], chemotherapy [9], hypersensitivity pneumonitis [10], and occupational exposure to asbestos and silicone [11]. Idiopathic PPFE has been classified as a rare idiopathic interstitial pneumonia (IIP) according to the American Thoracic Society (ATS)/European Respiratory Society (ERS) classification of IIPs [3]. Many reports on the clinical or imaging features of idiopathic PPFE have already been published, and some diagnostic criteria have been proposed [12].

The pathogenesis of PPFE has not yet been identified. We reported that alveolar epithelial denudation (AED) was prominent in a case of PPFE caused by hematopoietic stem cell transplantation [13]. This AED occurred at the transition zone between the affected area and adjacent normal parenchyma. Moreover, there were many lymphocytes in the area of AED, which suggested that donor lymphocytes could directly damage the alveolar epithelium and cause AED. Therefore, AED may be the earliest histopathological change in PPFE. We hypothesized that AED might lead to subsequent alveolar collapse, suggesting that it may be an important event in the pathogenesis of PPFE. Furthermore, we hypothesized that AED was a major factor involved in the pathogenesis of PPFE, other than in hematopoietic stem cell transplantation. It was also reported in IPF that apoptosis is induced by alveolar epithelial dysfunction, and localized epithelial denudation leads to the formation of fibroblastic foci [14,15]. 

Therefore, we aimed to morphologically investigate AED in PPFE and IPF and compare the frequency of AED between the two. Normal lung tissue was included as a control.

## 2. Materials and Methods

### 2.1. Study Subjects

This study was a multicenter, retrospective, cross-sectional study. From January 2013 to March 2019, 26 patients diagnosed with PPFE by surgical lung biopsy or autopsy at our institution with histopathological criteria described in the guidelines [3,12] were enrolled as the PPFE group. In addition, between January 2016 and March 2019, 30 patients diagnosed with UIP/IPF by surgical lung biopsy and 29 patients with no underlying respiratory disease who underwent partial lung resection for metastatic lung tumors were enrolled as the IPF and control groups, respectively. The sample size of this comparative group was determined by performing Welch’s t-test with a detection power of 80% and a significance level of 5% using the results of the preliminary study. For these three groups, we obtained the patient background, blood test results, and pulmonary function test results at the time of biopsy or autopsy. Paraffin blocks for each case were retrieved from the archives of the participating institutions.

### 2.2. Histopathological Assessment

A single representative slide from each case was selected by two expert pulmonary pathologists blinded to the clinical information and diagnosis. Hematoxylin and eosin (H&E) and immunohistochemical staining for cytokeratin AE1/AE3 were performed on sections from each case. The sections were scanned at 40× using an Ultra Fast Scanner (Phillips, Amsterdam, Netherlands), and whole-slide images (WSI) were generated. These WSIs were evaluated by six pathologists comparing H&E staining and pan-cytokeratin AE1/AE3 (Agilent, Santa Clara, CA, USA) immunohistochemical staining. To minimize interobserver variation, initial training on the evaluation of AED was given to all participants by two expert pulmonary pathologists. For that, six cases including two WSIs from each study group were reviewed and discussed to reach an agreement on AED evaluation by all pathologists. In addition, the proportion of the pathologists in charge of each group was assigned equally to minimize bias.

**Figure 1:**
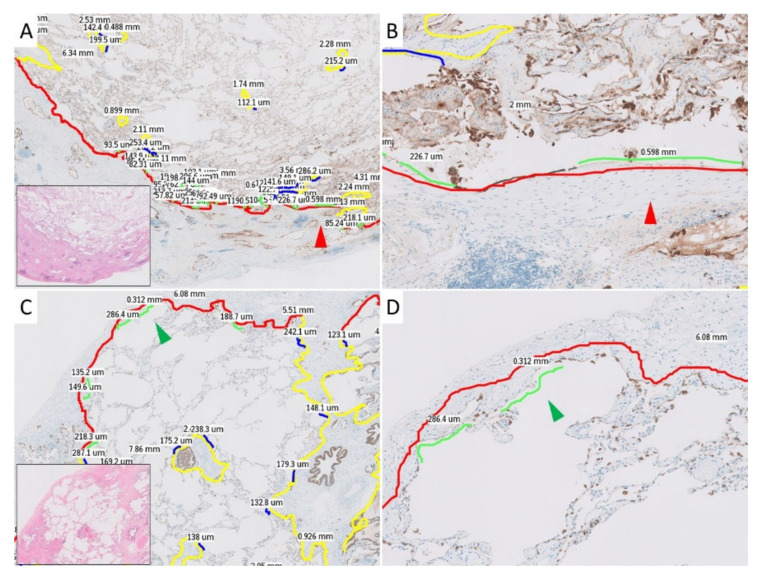
Histopathological images with annotations used in the current study. The red and green arrowheads each indicate the same location. The details of the colors in the annotation lines are as follows: red, subpleural zone; yellow, paraseptal zone; green, alveolar epithelial denudation (AED) in the subpleural zone; blue, AED in the paraseptal zone. (**A**): Annotated pleuroparenchymal fibroelastosis (PPFE) case in middle power view. (**B**): Annotated PPFE case in high power view. (**C**): Annotated idiopathic pulmonary fibrosis (IPF) case in middle power view. (**D**): Annotated IPF case in high power view.

First, with low magnification at 10×, the subpleural and paraseptal zones were identified and marked using annotation tools in the IMS viewer (Philips). The subpleural zone was defined as the border between the normal alveolar zone and the subpleural part of the subpleural connective tissue, including subpleural fibroelastosis and dense fibrosis. The paraseptal zone was defined as the border between the normal alveolar zone and the connective tissue of the interlobular septum of the bronchovascular bundle. Each area was annotated, and their lengths were measured on WSI. Then, with medium magnification at 100×, sites of AED in subpleural and paraseptal zones were identified, annotated, and their lengths were measured on WSI. H&E-stained slides were used to identify fibrous lesions and distinguish subpleural and paraseptal zones, and AE1/AE3 staining was used to define AED. Finally, the denudated ratios were calculated from the subpleural zones, paraseptal zones, and their measured sum by dividing the length of the AED areas by the overall lengths of the zones. The ratios (expressed as percentages) were compared between groups. The characteristics and longest range of AED were also recorded. Examples of annotated WSIs are shown in Figure 1.

### 2.3. Statistical Analysis

All numerical data are presented as median values with a 25–75% interquartile range. Statistical significance of the difference between two or three groups was analyzed using the Wilcoxon rank-sum test, Mann–Whitney U test, or Fisher’s exact test, where applicable. Statistical significance was defined as *p* < 0.05, and all statistical analyses were performed using JMP 14.0 (SAS Institute, Cary, NC, USA).

## 3. Results

### 3.1. Patient Characteristics

Patient characteristics for each group are shown in Table 1. Twenty-six patients in the PPFE group, 30 in the IPF group, and 29 in the control group were evaluated. Compared to the other two groups, the IPF group included more males (83.3%, *p* = 0.012) and smokers (73.3%, *p* = 0.001). Blood biochemistry tests showed that Krebs von Lunge-6 (KL-6) in the PPFE group was 424 IU/mL, which was lower than that in the IPF group (1183 IU/mL; *p* < 0.001). However, surfactant protein-D (SP-D) did not differ between the PPFE group (221 IU/L) and the IPF group (222 IU/L). In pulmonary function tests, the PPFE group had a significantly lower percentage of forced vital capacity (%FVC) of 70.9% (*p* = 0.016) and a significantly higher residual volume/total lung capacity (RV/TLC) of 43.1% (*p* < 0.001) compared to the IPF group, which was consistent with previous reports [12,16]. The IPF group had a significantly lower percentage of diffusing capacity of the lung for carbon monoxide (%DLCO) of 65.3% compared to the other two groups (*p* = 0.02).

### 3.2. Histological Findings

**Figure 2:**
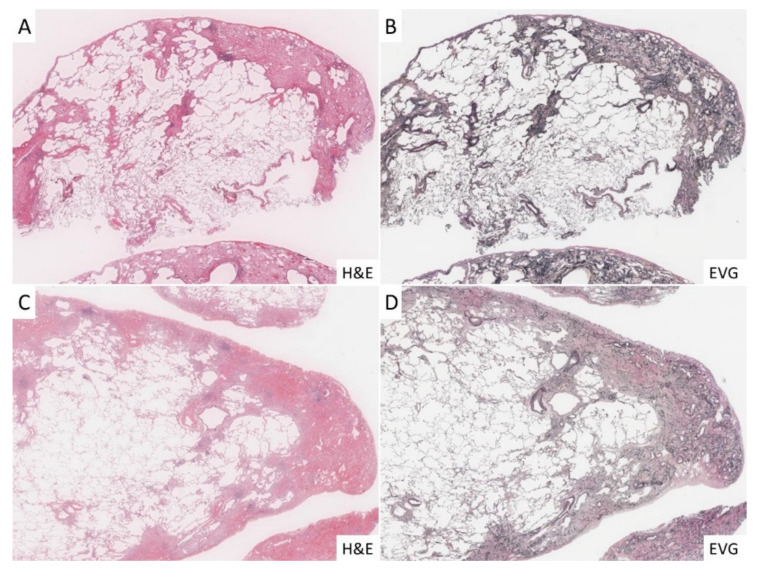
Histopathological findings in the cases investigated for the current study. (**A**,**B**): In the PPFE group, fibroelastosis was significant under the pleura, and there was marked increase in elastic fibers with EVG staining. (**C**,**D**): In the IPF group, the development of dense fibrosis and fibroblastic foci was significant in subpleural and interlobular septa, and lung architecture destruction was highlighted with EVG staining.

The histopathological images of the three groups were reviewed in all cases and confirmed to be consistent with the diagnosis. Briefly, all cases in the PPFE group had fibroelastosis, a fibrotic lesion consisting mainly of a dense growth of elastic fibers in the subpleural space (Figure 2A,B), and all IPF cases had dense fibrotic changes with architectural distortion in the subpleural and interlobular septa (Figure 2C,D). In the control group, we did not observe histopathological findings similar to those seen in the PPFE and IPF groups.

**Figure 3:**
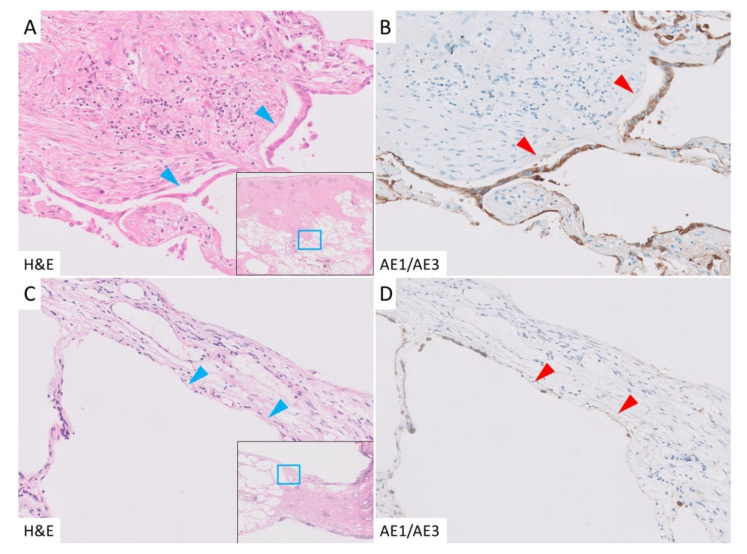
Epithelial denudation in the PPFE group. (**A**): Alveolar epithelial denudation (AED) was detected in the border between the subpleural fibroelastosis and normal lung (blue arrowhead). (**B**): Cytokeratin AE1/AE3 immunohistochemical staining. The denudated epithelium and detached surface were highlighted in this staining (red arrowhead). (**C**,**D**): AED was also seen in the subpleural area without fibroelastosis (arrowhead).

**Figure 4:**
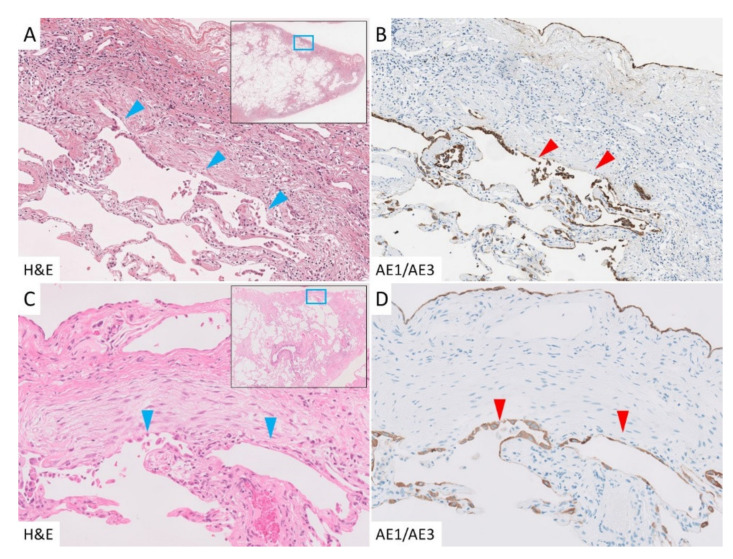
Epithelial denudation in the IPF group. (**A,B**): Dense fibrosis and abrupt changes in the normal lung were seen, with noticeable AED in those areas (arrowhead). (**C,D**): Fibroblastic foci were well observed, some of which were also accompanied by AED, but most were covered with regenerating epithelium (arrowhead).

In cases of PPFE, the border between subpleural fibroelastosis and the normal lung tissue (subpleural zone) was well defined, and there was conspicuous AED in the area, which was highlighted by AE1/AE3 immunohistochemical staining (Figure 3A,B). Unlike in the subpleural zone, fibroelastosis was rarely observed in the interlobar septa or the area around the bronchovascular bundle (paraseptal zone), but AED was also observed in the paraseptal zone. AED was evident in areas of fibroelastosis, but interestingly, it was also observed in areas lacking fibroelastosis (Figure 3C,D). In the IPF group, AED was observed at the border between the dense fibrosis and normal lung tissue. In contrast to the PPFE group, AED was rarely found in the unaffected areas. In areas where fibroblastic foci had formed, AED was seen occasionally (Figure 4A,B), but most were covered with regenerating epithelium (Figure 4C,D). In the control group, only a few AEDs were observed.

### 3.3. Measurement of Alveolar Denudation

**Figure 5:**
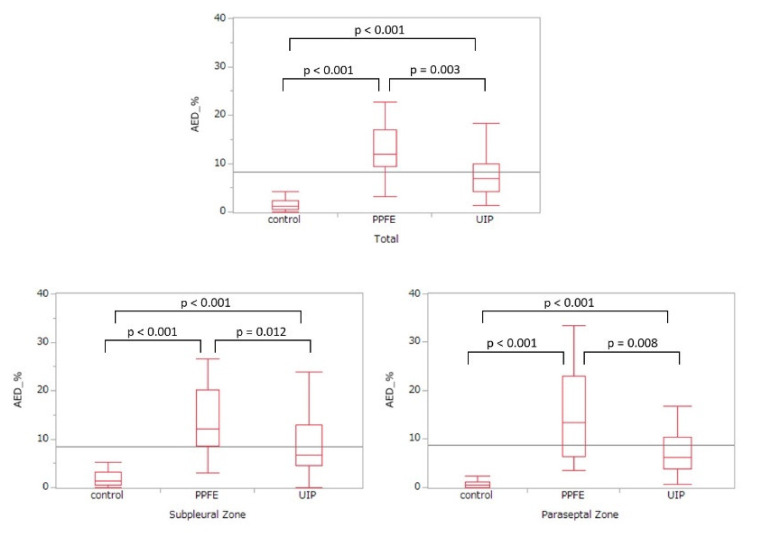
Percentage of alveolar epithelial denudation in each group.

The denudated ratios were calculated for each of the three groups (Table 2 and Figure 5) from the total length and AED lengths of the subpleural and paraseptal zones. The median percentage of AED in the PPFE group was 12.01%, indicating significantly higher denudation compared to the IPF group (*p* = 0.003), which was 6.84%, and the control group (*p* < 0.001), which was 1.19%. Compared to that in the IPF and control groups, AED in the PPFE group was higher in the paraseptal and subpleural zones. AED was significantly higher in the IPF group than in the control group (*p* < 0.001), but less than that in the PPFE group.

The lengths of continuous AEDs were examined, and a maximum of 3.52 mm and a median of 0.73 mm were found in the PPFE group. The IPF group showed a maximum of 1.44 mm and a median of 0.58 mm, which was not significantly different from the PPFE group (*p* = 0.08) (Supplementary Table S1).

## 4. Discussion

In this study, we showed that AED in PPFE was statistically more extensive than in IPF or normal lung tissue. It is known that AED precedes fibrin deposition and hyaline membrane formation in diffuse alveolar damage, which is a major histological pattern of acute lung injury. Fukuda et al. reported that in 16 cynomolgus monkeys in which lung injury was induced by intravenous injection of paraquat, AED occurred as the first histopathological change 2–3 days after injection, which lead to subsequent fibroblast induction and fibrosis [17]. This report showed that AED was the first histopathological change in acute lung injury, followed by inefficient repair and uncontrolled activation and proliferation of (myo)fibroblasts [18]. Moreover, the death of alveolar epithelial cells and loss of epithelium were reported as the first histopathological changes in IPF that lead to subsequent development of alveolar collapses and fibroblastic foci [19]. Fibroelastosis is a common histological reaction in many diseases with pulmonary collapse, such as middle lobe syndrome, apical cap, round atelectasis, and the central portion of noninvasive adenocarcinoma [20,21]. Alveolar collapse has various causes, and AED is a predicted cause of alveolar collapse. Our observations in post-transplant PPFE [13] also suggested that AED was a major factor in the etiology of PPFE. However, there are no reports on the pathogenesis of PPFE. The present study is the first to show that AED may be involved in the development of PPFE.

Another important point in the current study is that AED was observed not only in the fibroelastotic area, but also in the subpleural zone, which was unaffected in PPFE. This is another reason why AED did not occur owing to PPFE lesions, but AED may be the first event leading to PPFE. AED was expected to cause a localized decrease in blood circulation and surfactant within the alveoli, leading to alveolar collapse. Alveolar collapse is an important trigger in the pathogenesis of PPFE, as recently reported by Kinoshita et al. [22], which supports our speculation.

Recent genome-wide association studies have shown that the alveolar epithelium of IPF patients was affected by genetic variation in telomerase [23] or MUC5B [24,25], which resulted in abnormalities, such as accelerated apoptosis and delayed regeneration of the alveolar epithelium [26]. In the current study, we showed that AED was significantly more prominent in patients in the IPF group than in the normal group, consistent with the above reports.

Our current study showed that AED was significantly more prominent in PPFE than in IPF. This proves the hypothesis that AED was a histopathological change in early-stage PPFE and IPF. Although AED developed more prominently in PPFE than in IPF cases, it did not lead to abnormal proliferation of mesenchymal cells, such as fibroblasts and myofibroblasts, or a marked increase in extracellular matrix deposition, as seen in IPF [18,27]. This suggested that PPFE and IPF are different diseases with pathologically distinct processes, although AED occurred similarly in both as an early microscopic manifestation. PPFE is a fibrotic disease in which lesions form mainly in the upper lobe, where physiological respiratory movements are limited to one third of the upper lobe compared to the lower lobe. Moreover, the physiological environment is different from IPF in which lesions form predominantly in the lower lobe. Alveolar collapse, which leads to fibrosis [19], may occur because of AED in PPFE. Moreover, AED in the unaffected area was seen in the PPFE group but not in the IPF group, which may be another reason why the pathological processes of PPFE and IPF are different. However, the present study did not provide a detailed comparison of AEDs in unaffected areas in the two groups. This is an issue for future study including biology.

To address this assumption, we considered the alternative hypothesis that PPFE-like lesions are formed in IPF cases with prominent AED. Notably, when the formation of PPFE-like lesions were observed in IPF cases with prominent AED, we considered that fibrosis in PPFE and IPF might consist of lesions of a similar spectrum. To investigate this hypothesis, we reviewed the computed tomography images of patients in the IPF group and compared the level of AED in a group of patients with a few PPFE-like lesions (*n* = 10) and a group without PPFE-like lesions (*n* = 19) (Supplemental Table S2). The results showed that the level of AED was similar in these two groups, suggesting that PPFE and IPF have the same initial histopathological changes of AED, but the subsequent fibrosis is different. This finding implies that PPFE and IPF have different pathogeneses. To date, no genetic events were reported in PPFE, in contrast to IPF [23,24,25], which supports our data. Further investigations, including molecular pathology of PPFE, are required to validate the above hypothesis. 

The following four points are mentioned as limitations of this study. First, this was a retrospective study, and the number of examined cases was limited. For example, one of the largest reported series of PPFE included only 62 patients [28]. On the other hand, considering the rarity of PPFE, we believe that the number of cases enrolled was sufficient to make meaningful conclusions. Second, the correlation of AED with prognosis and disease progression after biopsy was not investigated, mainly because the present study emphasized histopathological parameters. Third, we could not examine the apoptosis of alveolar epithelial cells, which should be more reliably evaluated in experimental studies aimed at investigating how AED may lead to lung fibrosis. Fourth, the present study did not compare the differences in AED between idiopathic PPFE and other causes.

## 5. Conclusions

We showed that PPFE frequently displayed alveolar denudation, which was significantly more prominent compared with IPF and normal lungs. The pathogenesis of AED in PPFE may be different from that in IPF.

## Figures and Tables

**Table 1:** Patient characteristics.

		PPFE Group	IPF Group	Control Group	*p*-Value *	*p*-Value **
Number		26	30	29		
Age		61 (56–69)	65 (60–68)	66 (58–73)	n/a	n/a
Gender: Male		17 (65.4%)	25 (83.3%)	13 (44.8%)	0.007	n/a
Smoker		14 (56.0%)	22 (73.3%)	8 (27.6%)	0.002	n/a
Blood biochemistry						
	KL-6 (IU/mL)	424 (299–675)	1182 (753–1642)	n/a	<0.001	<0.001
	SP-D (IU/L)	221 (123–353)	222 (141–321)	n/a	n/a	n/a
Pulmonary function test						
	FVC (%pred.)	70.9 (61.9–85.0)	88.2 (70.1–104.6)	109.3 (96.8–126.8)	<0.001	0.016
	FEV_1_/FVC (%)	92.9 (82.7–97.3)	81.9 (72.6–86.5)	73.0 (64.5–78.1)	<0.001	0.001
	RV/TLC (%)	43.1 (35.5–49.4)	30.4 (23.6–34.5)	34.4 (31.7–38.5)	<0.001	<0.001
	%DL_CO_ (%pred.)	83.6 (54.1–89.4)	65.3 (55.6–84.3)	87.3 (74.4–99.2)	0.004	n/a

DLCO, diffusing capacity of the lung for carbon monoxide; FEV1%, forced expiratory volume in 1 s; FVC, forced vital capacity; IPF, idiopathic pulmonary fibrosis; KL-6, Krebs von Lunge-6; PPFE, pleuroparenchymal fibroelastosis, RV, residual volume; SP-D, surfactant protein D; TLC, total lung capacity. * among three groups; ** PPFE vs. IPF.

**Table 2:** Percentage of alveolar epithelial denudation in each group.

	PPFE Group	IPF Group	Control Group	*p*-Value *	*p*-Value **	*p*-Value ***
Total	12.01 (9.41–16.92)	6.84 (4.26–9.93)	1.15 (0.56–2.37)	0.003	<0.001	<0.001
Subpleural zone	12.08 (8.63–20.11)	6.68 (4.61–12.95)	1.38 (0.52–3.18)	0.012	<0.001	<0.001
Paraseptal zone	13.41 (6.34–22.95)	6.25 (3.82–10.41)	0.45 (0.06–1.19)	0.008	<0.001	<0.001

IPF, idiopathic pulmonary fibrosis; PPFE, pleuroparenchymal fibroelastosis. * PPFE vs. IPF. ** PPFE vs. Control. *** IPF vs. Control.

## Data Availability

The data presented in this study are available on request from the corresponding author. The data are not publicly available due to recommendations from the Institutional Review Board based on ethical considerations.

## Supplementary Materials

The following are available online at https://www.mdpi.com/2077-0383/10/5/895/s1, Table S1: Maximum length (μm) of continuous epithelial denudation in each group, Table S2: Percentage of epithelial denudation in the IPF group with or without PPFE-like lesions.
