# Supplementary material for: Alveolar Epithelial Denudation Is a Major Factor in the Pathogenesis of Pleuroparenchymal Fibroelastosis
Source: J Clin Med. 2021 Feb 24;10(5):895. doi: 10.3390/jcm10050895 (PMC7956653; doi:10.3390/jcm10050895)
Supplement: Supplementary file 1 [file jcm-10-00895-s001.pdf]

Journal of
Clinical Medicine

MDPI

Supplementary file

Alveolar Epithelial Denudation is a Major Factor in the Pathogenesis of Pleuroparenchymal Fibroelastosis

Yoshiaki Zaizen [1,2], Yuri Tachibana [1], Yukio Kashima [3], Andrey Bychkov [1,4], Kazuhiro Tabata [5], Kyoko Otani [6], Yoshiaki Kinoshita [7], Yasuhiko Yamano [8], Kensuke Kataoka [8], Kazuya Ichikado [9], Masaki Okamoto [2], Tomoo Kishaba [10], Remi Mito [11], Koichi Nishimura [12], Mari Yamasue [13], Kazuki Nabeshima [14], Kentaro Watanabe [15], Yasuhiro Kondoh [8] and Junya Fukuoka [1,4,*]

1 Department of Pathology, Nagasaki University Graduate School of Biomedical Sciences, 1-7-1 Sakamoto, Nagasaki 852-8501, Japan; zaizen_yoshiaki@med.kurume-u.ac.jp (Y.Z.); y.tachibana19890221@gmail.com (Y.T.); bychkov.andrey@kameda.jp (A.B.)
2 Division of Respirology, Neurology and Rheumatology, Department of Medicine, Kurume University School of Medicine, 67 Asahi-machi, Kurume, Fukuoka 830–830-0011, Japan; okamoto_masaki@med.kurume-u.ac.jp
3 Department of Pathology, Hyogo Prefectural Awaji Medical Center, 1-1-137 Shioya, Sumoto, Hyogo 656-0021, Japan; yksmn0504@yahoo.co.jp
4 Department of Pathology, Kameda Medical Center, 929 Higashi-cho, Kamogawa, Chiba 296-8602, Japan
5 Division of Pathology, Department of Oncology, Kagoshima University Graduate School of Medical and Dental Sciences, 8-35-1 Sakuragaoka, Kagoshima 890-8544, Japan; tabatak.kufm@gmail.com
6 Department of Pathology, Yodogawa Christian Hospital, 1-7-50 Kunijima, Higashiyodogawa-ku, Osaka 533-0024, Japan; o.kyoko303@gmail.com
7 Department of Respiratory Medicine, Fukuoka University Chikushi Hospital, 1-1-1 Zokumyouin, Chikushino, Fukuoka 818-8502, Japan; y3kinoshita@gmail.com
8 Department of Respiratory Medicine and Allergy, Tosei General Hospital, 160 Nishioiwake, Seto, Aichi 489-8642, Japan; yaya0630g@gmail.com (Y.Y.); kataoka@tosei.or.jp (K.K.); konyasu2003@yahoo.co.jp (Y.K.)
9 Department of Respiratory Medicine, Saiseikai Kumamoto Hospital, 5-3-1 Chikami, Minami-ku, Kumamoto 861-4193, Japan; kazuya-ichikado@saiseikaikumamoto.jp
10 Department of Respiratory Medicine, Okinawa Chubu Hospital, 281 Miyazato, Uruma, Okinawa 904-2293, Japan; kishabatomoo@gmail.com
11 Department of Respiratory Medicine, Kumamoto University Hospital, 1-1-1 Honjou, Chuou-ku, Kumamoto 860-8556, Japan; candypinkcolor@yahoo.co.jp
12 Department of Respiratory Medicine, National Center for Geriatrics and Gerontology, 7-430 Morioka-chou, Oobu, Aichi 474-8511, Japan; koichi-nishimura@nifty.com
13 Department of Respiratory Medicine, Oita University Hospital, 1-1 Idaigaoka, Hasamamachi Yufu, Oita 879-5593, Japan; sai-mari@oita-u.ac.jp
14 Department of Pathology, Fukuoka University School of Medicine and Hospital, 7-45-1 Nanakuma, Jonan-ku, Fukuoka 814-0180, Japan; kaznabes@fukuoka-u.ac.jp
15 Department of Respiratory Medicine, Nishi Fukuoka Hospital, 3-18-8 Ikinomatsubara, Nishi-ku, Fukuoka 819-8555, Japan; watanabe@fukuoka-u.ac.jp
* Correspondence: fukuokaj@nagasaki-u.ac.jp; Tel.: +81-95-819-7055; Fax: +81-95-819-7056

Supplemental Table S1. Maximum length (μm) of continuous epithelial denudation in each group.

	PPFE Group	IPF Group	Control Group	*p*-Value*	*p*-Value**	*p*-Value***
Total	730 (365–1465)	580 (398–843)	250 (100–400)	0.086	<0.001	<0.001
Subpleural zone	715 (340–1278)	475 (298–723)	210 (100–280)	0.077	<0.001	<0.001
Paraseptal zone	425 (263–688)	465 (290–638)	145 (20–320)	0.774	<0.001	<0.001

IPF, idiopathic pulmonary fibrosis; PPFE, pleuroparenchymal fibroelastosis. * PPFE vs. IPF. ** PPFE vs. Control. *** IPF vs. Control.

Supplemental Table S2. Percentage of epithelial denudation in the IPF group with or without PPFE-like lesions.

	**With PPFE-like lesion**	**Without PPFE-like lesion**	***p*-value**
Number	10	19	
Total	7.77 (3.23–16.50)	6.30 (5.33–9.78)	0.819
Subpleural zone	6.64 (3.49–15.05)	6.57 (4.88–11.86)	0.819
Paraseptal zone	8.18 (2.84–17.74)	5.89 (4.31–7.80)	0.819
